# In the Law Courts

**Published:** 1894-12-15

**Authors:** 


### In the Law Courts.

### SURGEONS AND NON-QUALIFIED MEN.

At the Yorkshire Autumn Assizes an action for slander was brought by Messrs. Jones and Castle, surgeons, against John Crannidge, farmer, veterinary surgeon, and bonesetter. Mr. Tindal Atkinson, Q.C., and Mr. Longstaffe were for the plaintiffs—Mr. Richard Field Castle, surgeon, and Mr. William Mackie Jones, surgeon, Wath-upon-Dearne—and Mr. Kershaw was for the defendant, Mr. John Crannidge, farmer and chemist, of Denaby. The plaintiffs, it was stated, had until recently been partners, and the defendant was a farmer who carried on business as a bonesetter, and was also an unauthorised but general practitioner. On April 2nd last a boy named Dawson sustained a serious fracture of the right forearm through being crushed between two tubs at Houghton Main Colliery. Dr. Jones was surgeon to the colliery, but as Dr. Castle lived close at hand the boy was taken to him. He set the arm temporarily owing to its swollen condition, and next day Dr. Jones and Dr. Castle re-set the arm together. On May 12th the splints were removed, and it was then found that the arm was somewhat stiff and swollen. The father was apparently not satisfied, but the medical gentlemen in question assured him that the arm would be all right. The father, however, went to the defendant Crannidge, who, after examining the arm, said that "from the way in which the arm was going on the lad would be a cripple for life. The arm," he said, "would never get better until it was re-broken and re-set." The defendant called in the assistance of his daughter and commenced to manipulate the boy's arm. The defendant having put the arm in splints of his own sent him off, charging 3s. 6d. for the operation and 1s. for some ointment. On the matter being noised abroad Dr. Jones went to the manager of the colliery and insisted upon a full inquiry. Dr. Blackburn, of Barnsley, was instructed by the proprietors of the colliery to hold an inquiry, and such examination took place on May 23rd, five days after the boy's arm was supposed to have been re-broken and re-set. In the opinion of Dr. Blackburn the statement that the arm had had to be re-broken and re-set was absolutely untrue. The bones were set and had joined, and at the request of Dr. Blackburn the boy lifted with the injured arm a 7lb. weight only five days after it was supposed to have been broken. Defendant was understood to say that his statement with reference to the plaintiffs was privileged, and under certain circumstances learned counsel said it might have been; but the jury must remember that defendant said he should have to break the arm, and that he said he had re-broken it when he had done nothing of the kind. Evidence was called in support of counsel's opening statement. Dr. Jones, Dr. Castle, Dr. Blackburn (Barnsley), Mr. Victor Horsley, F.R.S., Mr. Mayo Robson, surgeon, Leeds, and Mr. Arthur Jackson, of Sheffield, gave evidence in support of the case for the plaintiffs—showing that the treatment that had been resorted to by the plaintiffs was the best that could be taken.

The case for the plaintiffs having closed, Mr. Kershaw, on behalf of the defendant, submitted that the occasion was a privileged one.

His Lordship: How do you make that out?

Mr. Kershaw: These people went to the defendant to consult him, and it was not material that the defendant was not a properly qualified man to give the advice sought.

His Lordship: I don't think the occasion is privileged in the sense you state. In view of the alleged defamatory statements made with regard to these persons I don't think that such statements would be privileged even if they had been made by a regular and properly qualified practitioner with regard to another. It is news to me that as soon as a man enters the room of a doctor any defamatory statement that may be made in that room by that doctor with regard to another is privileged.

Mr. Atkinson submitted that in order that the occasion might be privileged there should be ground for making the statement complained of. In the present case there was no such justification.

The Judge ruled that in the present instance, so far as the case had gone, there was no privilege in the sense argued.

The case for the defence was afterwards proceeded with. The defendant himself, John Crannidge, was called, and stated what had taken place on the occasion of the visit of the boy Dawson and his father. Witness and daughter re-broke the arm, and he afterwards bandaged the arm in proper form. He had many patients in the bonesetting way, and his daughter assisted him.

Mr. Atkinson, Q.C.: What is your average number of patients a day?

Defendant: It varies. Sometimes I have 30 to 35, sometimes 20, and now and again I have only four or five.

Mr. Atkinson, Q.C.: Will you explain what all the letters mean after your name? What does "R.C., M.P.C., V.S." mean? (Laughter.)

Defendant: R.C. means Registered Chemist; M.P.C. means Member of the Pharmaceutical Conference; and V.S. means Veterinary Surgeon. I have set bones of animals many a time.

Mr. Atkinson: Have you gone through any course of surgery?

Defendant: I have been with medical men. (Laughter.) In their company.

Miss Crannidge was afterwards called, and bore out her father's evidence.

Mr. Atkinson: Perhaps you set bones yourself?

Witness: Sometimes I do when my father is out.

Mr. Atkinson: Have you gone through any surgical course yourself? Have you ever been even a hospital nurse?

Witness: No.

Mr. Atkinson: And your charge is 3s. 6d.

Witness: It varies. (Laughter.)

Mr. Atkinson: According to the ability of the patients to pay?

Witness: Yes; and some people never pay at all. (Laughter.)

The jury retired to consider their verdict. On returning into Court after an absence of about half an hour, they found that the words complained of had been used by the defendant, that they were slanderous, that they were not true, and according to the learned Judge's ruling as to whether Crannidge was actuated by malice, they were inclined to answer in the affirmative. Finally, they believed that the defendant's wish was to exalt his office so as to damage the plaintiffs. The damages they assessed at £30.

His Lordship gave judgment for the plaintiffs for the amount stated, and certified for a special jury.

